# Identify and validate RUNX2 and LAMA2 as novel prognostic signatures and correlate with immune infiltrates in bladder cancer

**DOI:** 10.3389/fonc.2023.1191398

**Published:** 2023-07-13

**Authors:** Yi Jin, Siwei Huang, Zhanwang Wang

**Affiliations:** ^1^Department of Radiation Oncology, Hunan Cancer Hospital, The Affiliated Cancer Hospital of Xiangya School of Medicine, Central South University, Changsha, Hunan, China; ^2^Key Laboratory of Translational Radiation Oncology, Department of Radiation Oncology, Hunan Cancer Hospital and The Affiliated Cancer Hospital of Xiangya School of Medicine, Central South University, Changsha, China; ^3^School of Humanities and Management, Hunan University of Chinese Medicine, Changsha, Hunan, China; ^4^Department of Oncology, Third Xiangya Hospital of Central South University, Changsha, China

**Keywords:** muscle-invasive bladder cancer, WGCNA, metastasis, prognosis, immune microenvironment

## Abstract

**Background:**

Muscle-invasive bladder cancer (MIBC) develops lymph node (LN) metastasis or distant metastasis, leading to recurrence and poor prognosis. The five-year survival rate of MIBC with LN or distant metastasis is only 8.1%; therefore, there is an urgent need to identify reliable biomarkers for prognosis and treatment regimen for patients with bladder cancer (BLCA).

**Methods:**

SEER database was used to select important clinical characteristics for MIBC. Then, weighted gene co-expression network analysis (WGCNA) was employed to identify differentially expressed genes (DEGs) to recognize significant co-expression modules by calculating the correlation between the modules and clinical data. Furthermore, Cox regression and lasso analysis were applied to screen prognostic hub genes and establish the risk predictive model. Bladder cancer cell lines (UMUC3 and 5637) were used for experimental validation *in vitro*.

**Results:**

Cox analysis of 122,600 MIBC patients showed that the N stage was the most important clinical factor. A total of 4,597 DEGs were calculated between N0 and N+ patients, and WGCNA with these DEGs in 368 samples revealed that expression of turquoise was positively and strongly correlated with the N stage. Eight genes were identified as important prognostic candidates using lasso regression based on Cox analysis and STRING database. Combining GEO datasets, literature, and clinical factors, we identified *LAMA2* and *RUNX2* as novel prognostic biomarkers. CCK8 assay showed that depletion of *LAMA2* or *RUNX2* significantly inhibited the proliferation of BLCA cells, and flow cytometry indicated that knockdown of *LAMA2* or *RUNX2* induced the apoptosis of BLCA cells. Transwell assay also showed that silencing of *LAMA2* or *RUNX2* weakened the migration and invasiveness of BLCA cells.

**Conclusions:**

We constructed a new eight-gene risk model to provide novel prognostic biomarkers and therapeutic targets for BLCA.

## Background

Bladder cancer (BLCA) ranks 10th among the most prevalent malignancies globally, with 573,000 new diagnosed cases and estimated 213,000 deaths (1). Among these BLCA patients, approximately 75% have diagnosed as non-muscle invasive BLCA (NMIBC), and the remaining 25% are muscle invasive BLCA (MIBC). Although NMIBC can usually be managed by intravesical treatment and transurethral resection, it may recur or develop aggressive MIBC (2). MIBC often results in lymph node or distant metastasis, leading to an unfavorable outcome (3). The 5-year survival rate of MIBC with lymph node or distant metastasis is only 8.1% (4). Currently, there is no effective treatment for metastatic BLCA. Therefore, early assessment of LN or distant metastases could represent an advantage to improve the prognosis.

In clinical practice, TNM (tumor, node and metastasis) staging are typically used for the prediction of prognosis for patients with BLCA. The overall survival (OS) of BLCA patients with positive lymph nodes and a higher clinical stage is poorer (5, 6). However, prognosis is based on inherent anatomical information, and predicting disease progression is difficult because of the biological heterogeneity of BLCA. Therefore, identification of reliable biomarkers is necessary for prognosis and designing treatment strategies of BLCA patients.

Weighted gene co-expression network analysis (WGCNA) is a novel systematic biological approach applied to clarify the connectivity of different gene clustering in a comprehensive network and evaluate the relationship of gene groups with diverse characteristics (7, 8). Compared to other calculation, WGCNA can be used to study hub genes closely associated with clinical phenotypes, providing a driving force for the discovery of new molecular biomarkers and therapeutic targets in BLCA (9–12).

In this study, we constructed a new eight-gene prognostic risk model in MIBC to predict the survival and prognosis of BLCA based on SEER, TCGA, and GEO datasets using WGCNA and lasso Cox regression methods. Moreover, we validated this model using external GEO datasets and identified the functions of the two hub genes through *in vitro* experimental assays, which providing prognostic biomarkers and therapeutic targets for BLCA.

## Methods and materials

### Data download and processing

SEER is a program that collects information on cancer patients in the USA, and is sponsored by the National Cancer Institute. We identified 122,600 MIBC patients and treated with cystectomy before 2016. Next, we used the Cox analysis to filter for important clinical factors that have an intimate relationship with overall survival. RNA sequencing from BLCA and clinical information, including gender, age, grade, tumor stage, and survival time, were downloaded from The Cancer Genome Atlas (TCGA) database (https://www.cancer.gov/tcga). Additionally, three datasets (GSE13507, GSE48075, and GSE48276) and the corresponding clinical information data were downloaded from the Gene Expression Omnibus (GEO) database (https://www.ncbi.nlm.nih.gov/geo/).

### WGCNA network construction

The “edgeR” R was employed to identify differentially expressed genes (DEGs) by R software 3.6.1 (13). DEGs were input to test their availability and construct a network based on the R package “WGCNA” (7). We included nine clinical characteristics: age, race, status, M grade, T grade, N grade, stage, height, and weight. In this study, we constructed an adjacency matrix used Pearson’s correlation coefficient and clustered the samples from TCGA, and drew a clinical characteristics-related sample clustering tree. After calculating and selecting an appropriate β value (β = 4), we transformed the adjacency matrix into a topological overlap matrix (TOM). Finally, based on TOM, we performed average‐linkage hierarchical clustering and module dendrograms to identify modules with a minimum gene dendrogram size of 30. The co-expression module is a collection of genes with high topological overlap similarity, and we identified significant clinical modules by calculating the correlation between the modules.

### Gene set enrichment analysis for biological function regression

After identifying the module that contained genes most related to important clinical characteristics, we continuously calculated DEGs in GSE48276 and GSE13507 to further filter them. To investigate the pathways, KEGG pathway analysis and GO biological processes by applying the clusterProfiler R package with a threshold *p*-value of <0.05, minimum count of 5, as mentioned in previously (14).

### Predictive model by lasso regression

The STRING database was applied to estimate protein–protein interactions. Based on STRING, we first performed a univariate Cox regression analysis to select prognostic genes related to each other. To enhance prediction accuracy and interpretability, lasso Cox regression analysis was carried out to construct prognostic models with the risk as follows:

Risk score = Expression_mRNA1_×Coefficient_mRNA1_ + Expression_mRNA2_×Coefficient_mRNA2_ +…Expression_mRNAn_×Coefficient_mRNAn_. According to the above model, patients were classified into high-risk (> median cutoff value) and low-risk groups. Subsequently, we implemented the Kaplan–Meier survival method to screen the availability of prognostic model, and a receiver operating characteristic (ROC) curve to evaluate the prediction accuracy of 1-, 3-, and 5-year OS. Additionally, we performed univariate and multivariate Cox analyses to distinguish clinicopathological parameters using the hazard ratio (HR) positively or negatively, and established a nomogram model using the package “rsm”. Continuous variables of the risk score and findings of Cox regression were included in our nomogram model. Finally, we validated and identified potential prognostic genes from the predictive model in GEO and TCGA datasets.

### Immune environment analysis in BLCA

Based on the expression of prognostic genes from lasso regression, patients were classified into diverse groups using the ConsensusClusterPlus R package with optimal k-means clustering (15). Subsequently, we applied the ESTIMATE algorithm (https://sourceforge.net/projects/estimateproject/) to estimate the ratio of immune stromal components in the tumor microenvironment (TME), including ESTIMATE, immune, and stromal scores (16). Additionally, CIBERSORT method was utilized to visualize the distribution of immune cell types. To further decipher the potential pathways related to TME, we obtained DEGs by comparing diverse groups. Gene enrichment analysis was performed on Metascape database, a powerful gene function annotation analysis tool (17).

### Cell culture and transfection

In this study, human BLCA cells (UMUC3 and 5637) were obtained from Procell company (Wuhan, Hubei, China). UMUC3 cells were maintained in Minimum Essential Medium (MEM; Procell, Wuhan, Hubei, China), and 5637 cells were maintained in RPMI-1640 Medium (1640; Procell, Wuhan, Hubei, China), supplemented with 1% penicillin-streptomycin liquid (Biosharp, Hefei, Anhui, China) and 10% fetal bovine serum (FBS; Procell, Wuhan, Hubei, China) and cultured in the humidified atmosphere with 5% CO_2_ at 37°C. Small interfering RNAs (siRNAs) of *RUNX2* and *LAMA2* were designed and synthesized from GenePharma Company (Shanghai, China). Lipofectamine 2000 (Invitrogen, Carlsbad, CA, USA) was used for transfection according to the manufacturer’s protocol. The transfection efficiency was confirmed by using quantitative reverse transcription polymerase chain reaction (qRT-PCR) assay.

### qRT-PCR

TRIzol reagent (15596026, Life Technologies, USA) was used to extract total RNA, following the manufacturer’s instructions. 1000 ng of RNA was subjected to synthesis cDNA by using the reverse transcription kit (RR037A, Takara, Dalian, China), then we performed qRT-PCR assay by using the TB Green Premix Ex Taq Kit (RR820A, Takara, Dalian, China). The 2^−ΔΔCt^ method was used for relative quantification of genes, and glyceraldehyde-3-phosphate dehydrogenase (GAPDH) gene was used as the internal control gene. The primer sequences and siRNAs are summarized in **Supplementary Table S3**.

### Cell counting kit-8 assay

A CCK8 assay kit (BS350B, Biosharp, Hefei, Anhui, China) was used to detect the proliferation of UMUC3 and 5637 cells. Approximately 3 × 10^3^ of UMUC3 or 5 × 10^3^ of 5637 cells were transfected with relevant siRNAs or scrambled negative control (NC) in triplicate and maintained in 96-well plates. Ten microliters of CCK8 reagent were added to each well, then the cells were incubated at 37°C for about 2 h. Optical density (OD) value at 450 nm was measured.

### Analysis of apoptosis by flow cytometry

A FITC Annexin V Apoptosis Detection Kit (556547; BD Biosciences) was used to detect the apoptosis rate of bladder cells. UMUC3 or 5637 cells at 1.0 × 10^5^ cells/mL density were seeded into 6-well plates, then cells were transfected with si-LAMA2, si-RUNX2, or NC within 24 h. After 72 h of transfection, cells were digested with EDTA-free trypsin and centrifuged, as described previously (18). The cell pellets were washed twice with phosphate buffer saline, then stained with 5 µL of propidium iodide and 5 µL of FITC Annexin V for 30 minutes in the dark. Finally, 400 µL of binding buffer was added to the cells and flow cytometry was used to observe the extent of apoptosis. Results were analyzed using FlowJo_V10.

### Transwell assays

Transwell chambers (3422, Coster, Corning, USA) coated without or with Matrigel matrix (356234, Corning, USA) were used to detect the migration and invasion of UMUC3 or 5637 cells. After 24 h of transfection with different siRNAs or NC, UMUC3 or 5637 cells were collected by trypsinization, and diluted at a density of 4 × 10^5^ cells/mL with serum-free medium. The upper compartment of chamber was plated with 200 μL of the diluted cell suspension. The lower compartment was supplemented with 600 μL of medium containing 20% FBS, and the cells were maintained in an incubator for 24 h.

Cells invading across membrane of the transwell were fixed with methanol for 15 min, then cells were stained with crystal violet solution (G1073, Solarbio, Beijing, China) for 15 another minutes. An inverted microscope (Olympus) was used to capture the images of stained cells. Five random fields were captured and used for counting the invading cells under the microscope.

### Statistical analyses

All bioinformatics analysis were performed by using R software 3.6.1. We assessed the relationship between the risk score and OS of patients with BLCA by using univariate or multivariate Cox proportional hazards regression analysis. All *in vitro* experimental data are represented as mean ± standard deviation (SD), and GraphPad Prism software (version 8.0; San Diego Inc., CA, USA) was used to perform data analysis. All experiments were independently repeated for three times, and statistical significance was set at *p*<0.05.

## Results

### Selection of prognostic clinical characteristics

A flowchart of experimental design and procedures is shown in **Supplementary Figure 1**. Out of 122,600 available patients, 92395 (75.4%) were men and 30205 (24.6%) were women, with a mean age of 69 years. The majority of patients (109091, 88.9%) were white. Patients who survived for less than three months were excluded, and a total of 115439 patients were enrolled for further analysis. In Cox analysis, age (HR = 1.06, 95% CI: 1.05–1.06, and *p*<0.001), race (HR = 0.98, 95% CI: 0.96–0.99, and *p* = 0.007), clinical stage (HR = 1.19, 95% CI: 1.16–1.22, and *p*<0.001), T (HR = 1.24, 95% CI: 1.21–1.27, and *p*<0.001), and N stage were prognostic clinical characteristics, particularly the N stage (HR = 1.38, 95% CI: 1.36–1.41, and *p*<0.001) (**Supplementary Figure S2**). We downloaded the clinical data and corresponding gene expression profiles from TCGA and GEO databases.

### Gene co-expression network construction of BLCA through WGCNA

**Figure 1:**
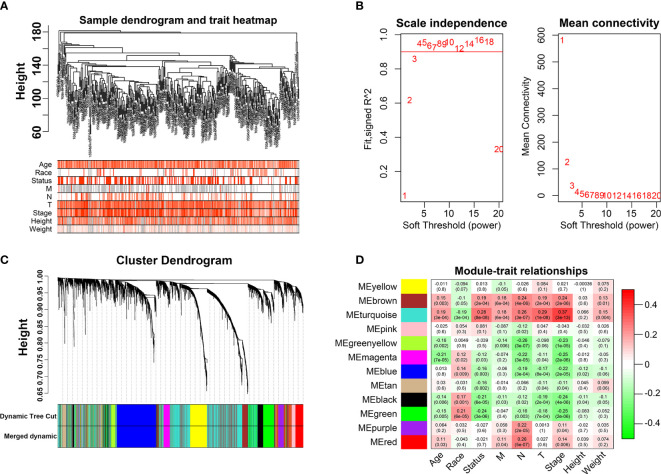
Weighted Co-expression Network Construction. **(A)** Dendrogram and traits heatmap for BLCA patients. **(B)** Network topology for different soft-thresholding powers. **(C)** Cluster dendrogram based on the dynamic tree cut algorithm. **(D)** Heatmap of the correlation between the clinical features and module eigengenes.

**Figure 2:**
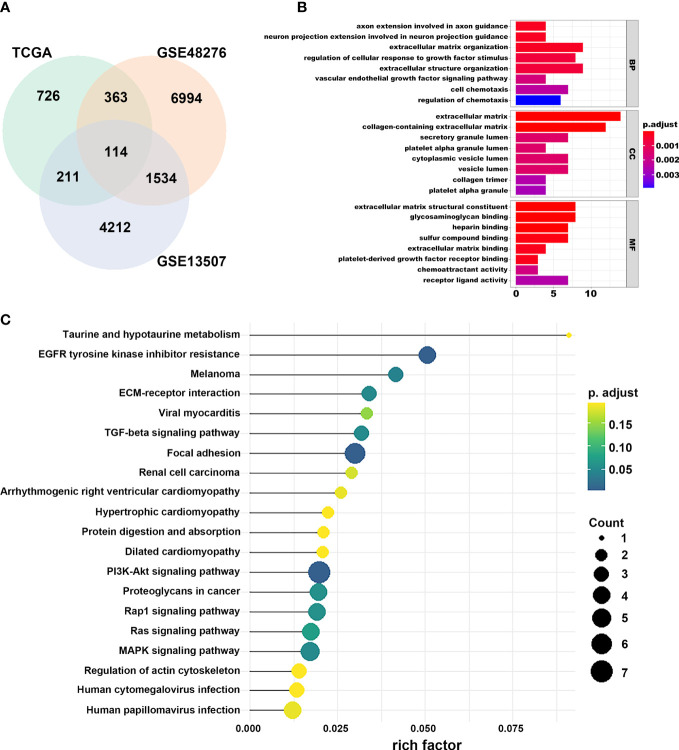
Selection of prognostic regulators and pathway function analysis. **(A)** Venn plot of regulators related to the N stage. **(B)** GO analyses of DEGs in turquoise module. **(C)** KEGG analyses of DEGs in turquoise module.

We first identified that the N stage was the most important clinical factor and evaluated the relationship between N stage and other clinical features (**Supplementary Figure S3**); therefore, we calculated and screened DEGs under the criteria of *p*<0.05 between N0 and N+ patients. A total of 4,597 genes were identified as DEGs in 368 BLCA samples for constructing the WGCNA network. Then, we merged the clinical features, including age, height, weight, race, status, T, N, and M stage to select key modules for DEGs and clinical factors of BLCA patients. The dendrogram and trait heatmap of those BLCA patients are shown in **Figure 1A**. β = 4 was set as the optimal soft threshold to construct a scale-free network, which ensured a high degree of scale independence (near 0.9) and low mean connectivity (close to 0) (**Figure 1B**). As shown in **Figure 1C**, we identified key gene co-expression modules based on the TOM. The heatmap with the interactions of co-expression modules indicated that the expression of turquoise, purple, red and brown modules was significantly and positively correlated with the N stage (**Figure 1D**), and the turquoise module presented a remarkable relationship with most clinical factors including age, race, T, N and M stages. Therefore, we determined this to be the key module. After dropping 1424 genes from the turquoise module, we calculated DEGs related to the N stage in GSE48276 and GSE13507, and believed that the overlapping genes might be significantly meaningful (**Figure 2A**). Finally, 114 genes remained, and GO and KEGG analyses revealed that these genes participated in EGFR tyrosine kinase inhibitor resistance, TGF-beta signaling pathways, and focal adhesion (**Figures 2B, C**).

### Building the prognostic risk model

**Figure 3:**
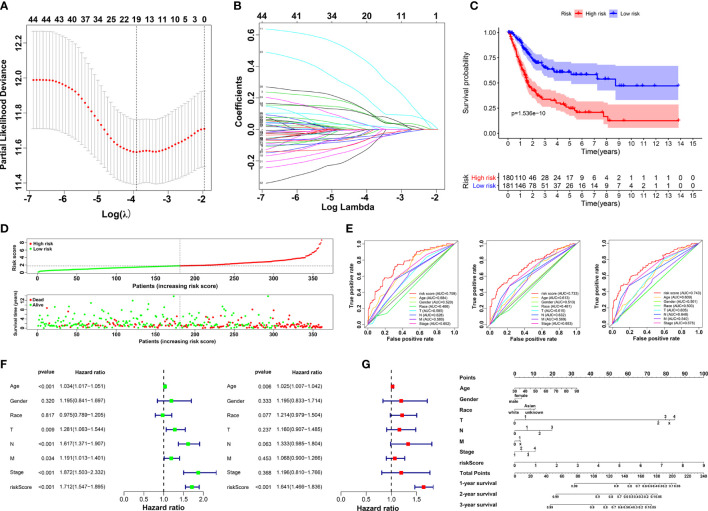
Construction of prognostic risk model. **(A, B)** Screening candidate crucial genes based on lasso Cox regression. **(C)** Overall survival analysis of low-risk and high-risk groups. **(D)** Distribution of risk scores, alive/dead status, and expression of 19 prognostic candidate genes. **(E)** The ROC curve of risk score and clinical traits in 1, 3 and 5 years. **(F)** Univariate (left) and multivariate Cox analysis (right) of clinical traits. **(G)** Nomogram construction of risk score and clinical traits.

Based on Cox analysis and STRING database, 45 genes were identified as important prognostic candidates for lasso regression. To develop a signature for prognosis prediction of BLCA, 19 genes (*C1QTNF6*, *DAB2*, *ESD*, *FKBP10*, *GAD1*, *INHBA*, *LAMA2*, *LGALS3*, *MPPED1*, *OLFML3*, *PCOLCE*, *RASD1*, *RGS12*, *RUNX2*, *TIMP2*, *TMEM151A*, *TSSK1B*, *VAT1*, and *VEGFA*) were obtained to build the risk model (**Figures 3A, B**). The Coefficient_mRNA_ of risk score and enrolled genes were shown in **Supplementary Table S1**. Patients were separated into low-risk and high-risk groups according to median cutoff of the risk score. Moreover, we found that patients may have significantly worse OS (*P* = 1.536e^-10^) with an increase in the risk score (**Figures 3C, D**). The ROC curve showed that the risk score had a better predictive ability than other clinical traits, with AUCs of 0.759, 0.733, and 0.743 at 1, 3, and 5 years compared with other factors (**Figure 3E**). Univariate and multivariate analyses further validated the risk score as an independent prognostic biomarker (**Figure 3F**). The constructed nomogram incorporating the risk score and other clinical traits is shown in **Figure 3G**, with C-Dex = 0.74, additionally, we drew the calibration to further depict the nomogram (**Supplementary Figure S4**).

### Characteristics of immune landscape in BLCA patients

**Figure 4:**
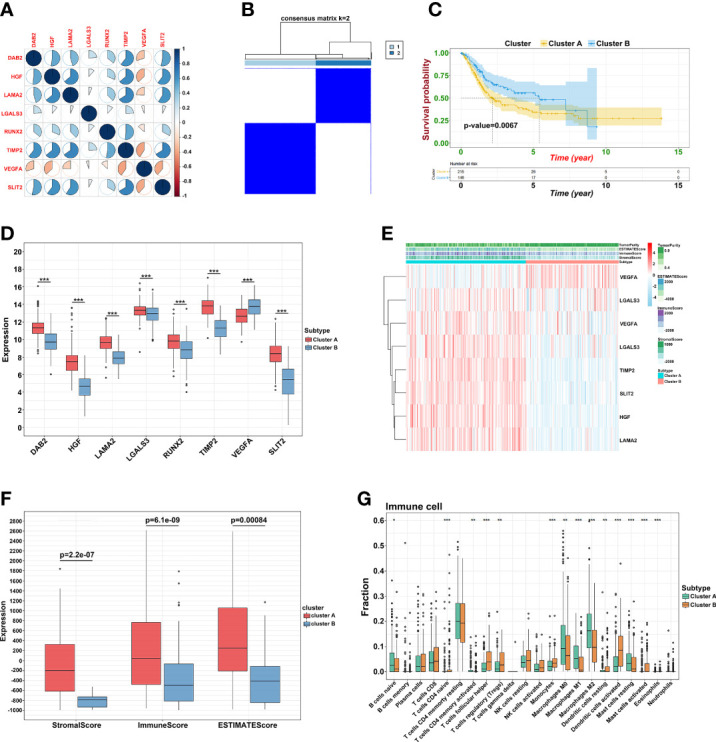
Prognostic signature construction. **(A)** Correlation between eight hub genes. **(B)** Consensus matrix for k = 2. **(C)** The KM plot of clusters A and cluster B **(D)** The expression of the eight regulatory factors in two clusters. **(E)** Heatmap of crucial genes from two clusters and ESTIMATE algorithm. **(F)** Different expression of Stromal, Immune and ESTIMATE score. **(G)** Different distribution of 22 TME infiltrating cells in two clusters (*p<0.05, **p<0.01, and ***p<0.001).

**Figure 5:**
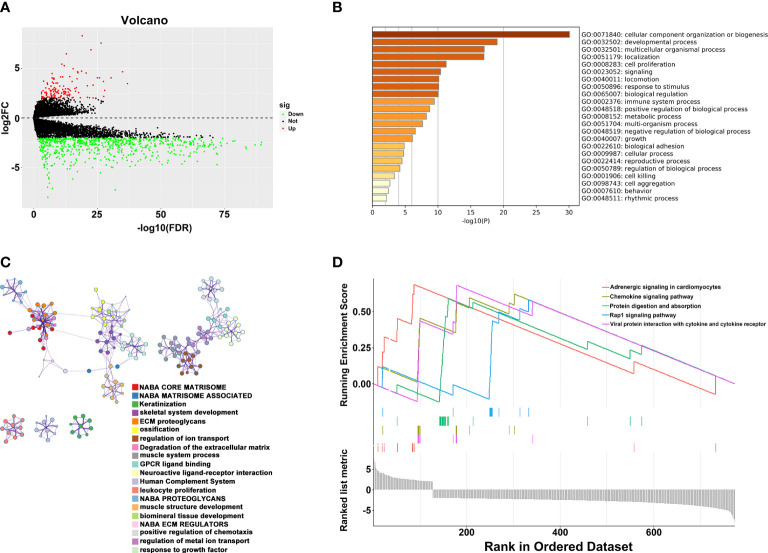
Relationship between two clusters. **(A)** Volcano plots of DEGs between two clusters. **(B)** Representative enriched terms of GO function. **(C)** The network colored by cluster-ID of GO function. **(D)** The GSEA analysis of DEGs between clusters 1 and 2.

To explore the potential mechanism of these genes in TME, we evaluated the relationship of 19 genes in STRING database and utilized Cytoscape to calculate all degree of nodes of the 19 regulators. Four genes (*C1QTNF6*, *GAD1*, *TMEM151A*, *and MPPED1*) were excluded based on STRING database, and six genes (*FKBP10*, *OLFML3*, *TSSK1B*, *ESD*, *RGS12*, and *VAT1*) were excluded according to cytoHubba from Cytoscape (DMNC, and clustering coefficient = 0) (**Supplementary Table S2**). Pearson’s correlation analysis was performed to determine the relationships among eight genes and risk score (*DAB2*, HGF, *LAMA2*, *LGALS3*, *RUNX2*, *TIMP2*, *VEGFA*, and *SLIT2*) (**Figure 4A** and **Supplementary Figure S5**). Based on the ConsensusClusterPlus R package, when the consensus matrix k value was equal to 2, there was the least crossover between TCGA samples. Therefore, we classified TCGA cohort into two groups by consensus expression (**Figure 4B**) and observed a significant difference between clusters A and B (**Figure 4C**). We also used the UMAP/PCA/tSNE reduction methods to re-evaluate the clusters (**Supplementary Figure S6**). Furthermore, we plotted a boxplot (**Figure 4D**) and heatmap (**Figure 4E**) to visualize the expression of the eight prognostic genes in the two clusters. To reveal the potential mechanism of these genes in TME, we used the ESTIMATE algorithm to apply Stromal, Immune, and ESTIMATE scores for BLCA samples. When compared to cluster B, the immune (*p* = 7.8e-12) and stromal scores (*p* = 0.0022) were significantly higher in cluster A, indicating that these clusters may participate in immune environment regulation (**Figures 4E, F**). We also evaluated the relationship between risk score and immune scores (**Supplementary Figure S7**). In addition, we utilized the CIBERSORT algorithm to analyze 22 different immune cell types among different clusters, which indicated that the levels of T cells CD4 naïve T cells regulatory (Tregs), T cells follicular helper, monocytes, dendritic cells activated, and dendritic cells resting in cluster A were obviously lower than those in cluster B. Moreover, the levels of T cells CD4 memory activated, T cells CD4 memory resting, macrophages M0, M1, and M2 were significantly higher in cluster A, suggesting that these clusters may strengthen or suppress the distribution of specific immune cell types, and potentially influence the response to immunotherapy (**Figure 4G**). Furthermore, 773 differentially expressed mRNAs were obtained by comparing clusters with |logFC (fold change) |≥1 and FDR<0.05, and volcano plots were drawn (**Figure 5A**) to visualize the distribution of DEGs. By analyzing Metascape database, we found that these shared mRNAs were predominantly enriched in tumor-related activities or pathways. For GO terms, mRNAs were enriched in collagen fibril organization, sensory organ development, and skeletal system development. For canonical pathways, mRNAs were enriched in NABA core matrisome and matrisome-associated pathways. For Reactome Gene Sets, mRNAs were enriched for keratinization and GPCR ligand binding (**Figures 5B, C**). GSEA was performed for further signaling pathway enrichment analysis, and in a comparison between clusters A and B, tumor-related pathways such as the IL-17 signaling pathway, drug metabolism cytochrome P450, and cytokine-cytokine receptor interaction were enriched (**Figure 5D**).

### Identification and verification of hub gene

**Figure 6:**
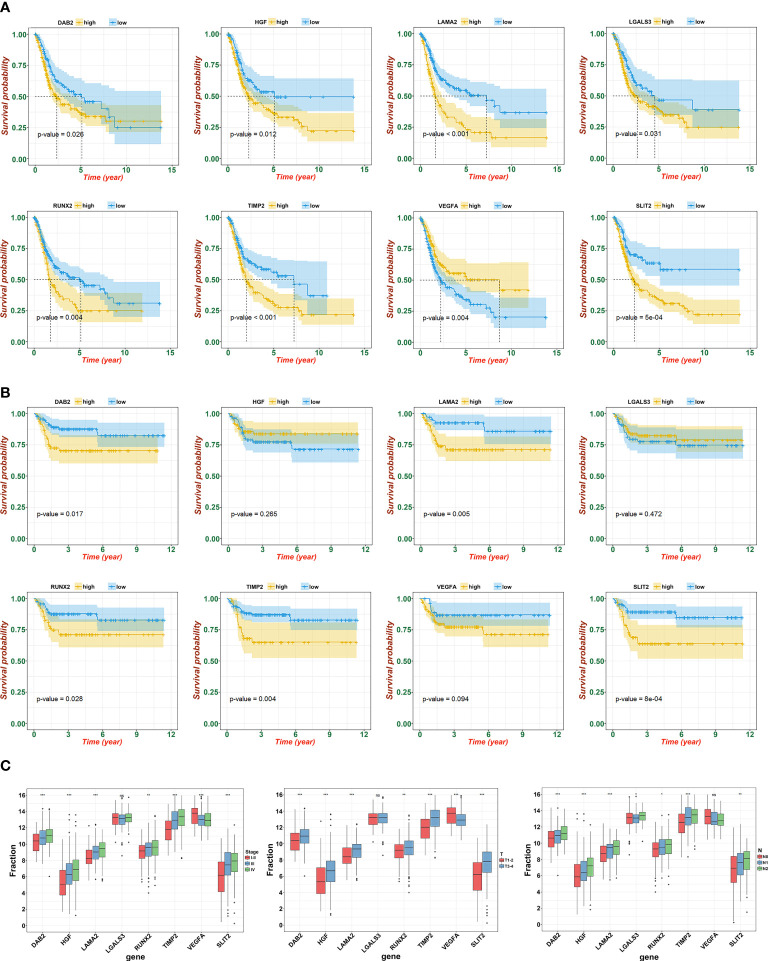
Identification and verification of hub gene. **(A)** The KM plot of hub genes and risk score in TCGA. **(B)** The KM plot of hub genes and risk score in GSE13507. **(C)** Expression of eight signatures in different T, N, and clinical stage (ns, not significant; *p<0.05, **p<0.01, and ***p<0.001).

To further determine the key prognostic immune-related genes, we continued to calculate the prognostic values of the eight genes in TCGA and GSE13507, and selected overlapping molecules that might be significantly meaningful. As shown in **Figure 6A** (TCGA) and **6B** (GSE13507), *DAB2*, *LAMA2*, *PCOLCE*, *RUNX2*, and *TIMP2* were successfully re-verified to induce poor OS. Furthermore, we observed that *LAMA2*, *DAB2*, and *TIMP2* had an intimate relationship with T, N, and clinical stages (**Figure 6C**).

### Effect of *LAMA2* or *RUNX2* silencing on growth, apoptosis, migration and invasion in BLCA cells

**Figure 7:**
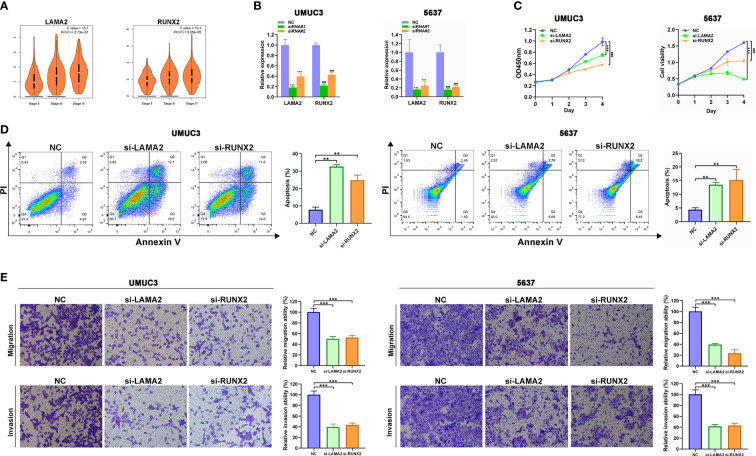
*LAMA2* and *RUNX2* promote the proliferation, migration, and invasiveness, and inhibit apoptosis of BLCA. **(A)** High expression of *LAMA2* and *RUNX2* are associated with advanced clinical stages through analyzing GEPIA database. **(B)** The silencing of *LAMA2* and *RUNX2* assessed using qRT-PCR. **(C)** CCK8 assay showing the effects of *LAMA2* or *RUNX2* knockdown on proliferation of UMUC3 cells. **(D)** Effects of *LAMA2* and *RUNX2* on apoptosis of BLCA as determined by flow cytometry. **(E)** Effects of *LAMA2* and *RUNX2* knockdown on migration and invasiveness of UMUC3 and 5637 cells, assessed using Transwell assay (**p<0.01, ***p<0.001, ^###^*p*<0.001, n = 3).

For further functional assays to verify the reliability and accuracy of our diagnostic model, we selected *LAMA2* and *RUNX2* after literature review, which have not been or rarely studied. In addition, we found that high expression level of *LAMA2* or *RUNX2* were associated with advanced clinical stages of BLCA (**Figure 7A**) by analyzing the GEPIA database. We designed and synthesized small interfering RNAs (siRNAs) targeting *LAMA2* and *RUNX2*. Compared with that in NC-transfected UMUC3 or 5637 cells, the expression of *LAMA2* or *RUNX2* (**Figure 7B**) was lower in UMUC3 or 5637 cells transfected with si-LAMA2-2 (named si-LAMA2) or si-RUNX2-1 (named si-RUNX2). Subsequently, CCK8 assay verified that silencing of *LAMA2* or *RUNX2* strikingly suppressed the growth rate of UMUC3 and 5637 cells (**Figure 7B**), indicating that both *LAMA2* and *RUNX2* play vital roles in promoting the proliferation of UMUC and 5637 cells. Flow cytometry analysis demonstrated that *LAMA2* or *RUNX2* could suppress the apoptosis of UMUC3 and 5637 cells (**Figure 7C**), indicating that the overexpression of *LAMA2* or *RUNX2* may promote BLCA proliferation by exerting an anti-apoptotic effect.

The transwell assay also demonstrated that the depletion of *LAMA2* or *RUNX2* significantly attenuated the migration and invasiveness of UMUC3 and 5637 cells (**Figures 7D, E**). Therefore, *LAMA2* and *RUNX2* could enhance the migration and invasiveness in BLCA cells.

## Discussion

BLCA is one of the most common urological tumors worldwide (1). Metastasis leads to poor prognosis of BLCA in patients, and currently has limited clinical curative effects (19, 20). However, the precision treatment of BLCA is gaining momentum, and its development requires the identification of hub genes closely related to BLCA. In this study, we utilized the Cox analysis to show that the N stage was the most important independent clinical factor. Moreover, interestingly, we found the age, and sex have intimate relationship with N stage with *p* < 0.001. As shown in **Supplementary Figure S2**, younger or black people were easily to get advanced N stage. Kim et al. (21) identified four key genes by utilizing 1320 genes to investigate progression-related genes in BLCA. Catto et al. (22) used artificial intelligence with immunohistochemical analysis to explore 11 progression-associated genes. However, these study models are only based on their clinical centers and without external or experimental validation, which limits their reproducibility and generalizability.

WGCNA is a system biology method used to describe correlation patterns among genes across microarray sequence data, and it is often used to link modules with external clinical features and identify important tumor genes (23). Deng et al. (24) identified *LRRC15*, *TRPM3*, *CYP1A2*, *CER1*, *ATF7*, *KCNIP1*, *PTPRJ*, and *GDF9* by constructing and estimating two normal and cancerous states, which were considered as the pivotal genes in bladder cancer. Luo et al. (25) screened *DACT3*, *TNS1*, and *MSRB3* using co-expression network analysis based on the WGCNA algorithm, which may provide novel therapeutic targets for BLCA patients with lymph node metastasis. However, these studies only compared cancerous and non-cancerous tissues or distant metastasis status. Considering that muscular-invasive bladder cancer is the most heterogeneous type of BLCA, we analyzed patients with MIBC using the SEER database and found that lymph node stage may be the most meaningful clinical factor affecting the prognosis of operable muscular-invasive bladder cancer. Tian (26) and Spradling (27) also reported that lymph node positivity is an independent predictor of recurrence and death in bladder cancer, which are similar to the conclusion of our study.

WGCNA has been widely used in multiple human cancers as a novel algorithm for clustering genes with the same function (28, 29). We used the WGCNA algorithm to identify genes closely related to lymph node metastasis and selected 19 core genes related to lymph node metastasis and prognosis of MIBC through lasso analysis. Furthermore, BLCA is a highly immunogenic tumor, and immunotherapy is widely used for patients with advanced stage, recurrence, metastasis, or multi-line treatment failure of BLCA. TME is an important marker for predicting the efficacy of immunotherapy, and disturbances in immune response in TME play a decisive role in the development of bladder cancer. The constituent immune cells of TME are an important part of tumor tissue. Lymph node staging is closely related to tumor immune response and immune microenvironment (30). To explore the potential mechanism of these 19 genes in TME, we assessed the relationship of these genes in STRING database and utilized the Cytoscape assay to calculate all degree of nodes of 19 regulators. Nine genes were identified as core prognostic factors related to lymph node metastasis and invasion, and used to evaluate their relationship with the immune microenvironment of BLCA (31, 32). Finally, we found that the different expression classifications of these nine genes formed different clusters of differences in immune scores. At the same time, immune cells such as T cells CD4 naïve, follicular helper T cells, and Tregs may be the key factors affecting the immune score and immune microenvironment.

To further determine core potential genes, we utilized GEO datasets and combined a variety of clinical data (T staging and TNM staging), and consulted the literature to select potential core genes that have never or seldom been reported in BLCA. Finally, we selected *LAMA2* and *RUNX2* to verify our hypotheses and the reliability of our research through *in vitro* experiments. We demonstrated that *LAMA2* and *RUNX2* acted as oncogenes to promote proliferation, migration, and invasion and prevent apoptosis of BLCA cells.

*LAMA2* encodes an alpha 2 chain and is a major component of basal laminae-α subunit of laminin, which plays important roles in normal and neoplastic tissues, including proliferation, adhesion, cell migration, and maintenance of cell shape and differentiation (33). *LAMA2* has recently been identified as a molecular marker of aggressive ependymoma (34), and a promoter of malignancy in glioblastomas (GBMs) through the maintenance of GBM stem cell compartment; therefore, it can be used as a molecular fingerprint and a possible therapeutic target for GBMs (35). However, suppression of *LAMA2* expression could promote the invasiveness of breast cancer cells (36), and low expression level of *LAMA2* predicted poor survival and higher recurrence rate in patients with hepatocellular carcinoma (37). Therefore, the function of *LAMA2* may be tumor-specific or dependent on the stage of oncogenesis. However, the role of *LAMA2* in bladder cancer has not been investigated. In this study, we first identified that the mRNA level of *LAMA2* was significantly associated with the prognosis and clinical stages of bladder cancer. Depletion of *LAMA2* significantly inhibited the proliferation, weakened invasiveness and migration, and promoted apoptosis of BLCA cells.

Runt-related transcription factor 2 (*RUNX2*), a member of the RUNX family, regulates developmental processes, including differentiation, apoptosis, proliferation, and cell lineage specification (38). The oncogenic functions of *RUNX2* were first identified in regulation of osteogenesis and strongly related to the progression of osteosarcoma (39). Subsequently, *RUNX2* was found to promote progression and bone metastasis in prostate and breast cancers. RUNX2 deficiency can attract myeloma cells and promote myeloma development at new bone sites by secreting metastatic cytokines and suppressing bone marrow immunity (40). However, the relationship between *RUNX2* and tumor immune microenvironment in BLCA was largely unknown. In this study, we first identified the role of *RUNX2* in BLCA and comprehensively assessed its profile in the immune landscape. Downregulation of *RUNX2* significantly inhibited the growth of BLCA cells, promoted their apoptosis, and weaken their migration and invasiveness, suggesting that *RUNX2* may be a prognostic biomarker and therapeutic target for BLCA. However, the biological roles of *LAMA2* and *RUNX2* and their specific molecular mechanism in BLCA remain unclear, which inspire us to further elucidate the potential underlying mechanism through molecular experiments and clinical trials.

## Conclusion

This study constructed an eight-gene risk signature model by using lasso regression analysis and WGCNA. The nine-gene risk signature owned meaningful performance in prognostic stratification in TCGA and GEO datasets. Furthermore, we comprehensively profiled immune cell infiltration and the landscape of tumor environment. Finally, two hub genes (*LAMA2* and *RUNX2*) were identified and successfully verified through *in vitro* experimental methods.

## Data availability statement

The datasets presented in this study can be found in online repositories. The names of the repository/repositories and accession number(s) can be found in the article/**Supplementary Material**.

## Ethics statement

The Institutional Review Board of the Ethics Committee of Hunan Cancer Hospital approved the consent procedure. No potentially identifiable human images or data ARE presented in this study.

## Author contributions

YJ designed the study. YJ analyzed, interpreted the data, wrote original draft. ZW wrote this manuscript. SH and ZW edited and revised the manuscript. All authors contributed to the article and approved the submitted version.

## Supplementary material

Supplementary Figure 1 Flow chart of the study without pictures.

Supplementary Figure 2 Cox analysis for patients with MIBC from the SEER database.

Supplementary Figure 3 The relationship between clinical features and N stage.

Supplementary Figure 4 The calibration of constructed nomogram.

Supplementary Figure 5 The KM plot of risk score in GSE13507 and GSE48276.

Supplementary Figure 6 The UMAP/PCA/tSNE reduction methods to re-evaluate the cluster.

Supplementary Figure 7 The correlation between risk score and immune scores.

Supplementary Table 1 The CoefficientmRNA of 19 prognostic biomarkers and the genelist of 45 enrolled genes.

Supplementary Table 2 The selection of hub genes according to the cytoHubba from Cytoscape.

Supplementary Table 3 The sequences of primers and siRNA used in this study.
