# Supplementary figures and images for: Identify and validate RUNX2 and LAMA2 as novel prognostic signatures and correlate with immune infiltrates in bladder cancer
Source: Front Oncol. 2023 Jul 13;13:1191398. doi: 10.3389/fonc.2023.1191398 (PMC10373733; doi:10.3389/fonc.2023.1191398)

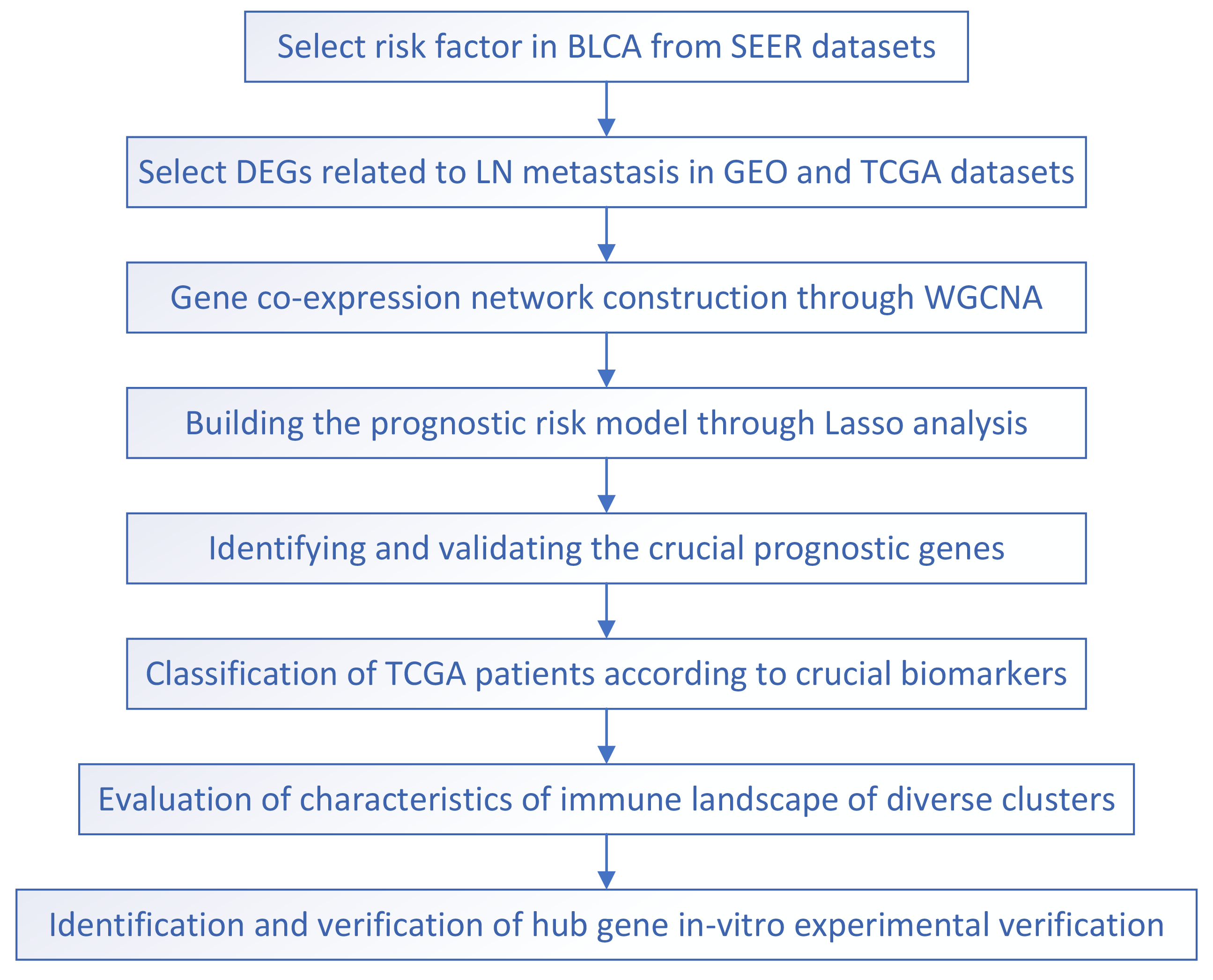

Supplement: Supplementary Figure 1 — Flow chart of the study without pictures. [file Image_1.tif]

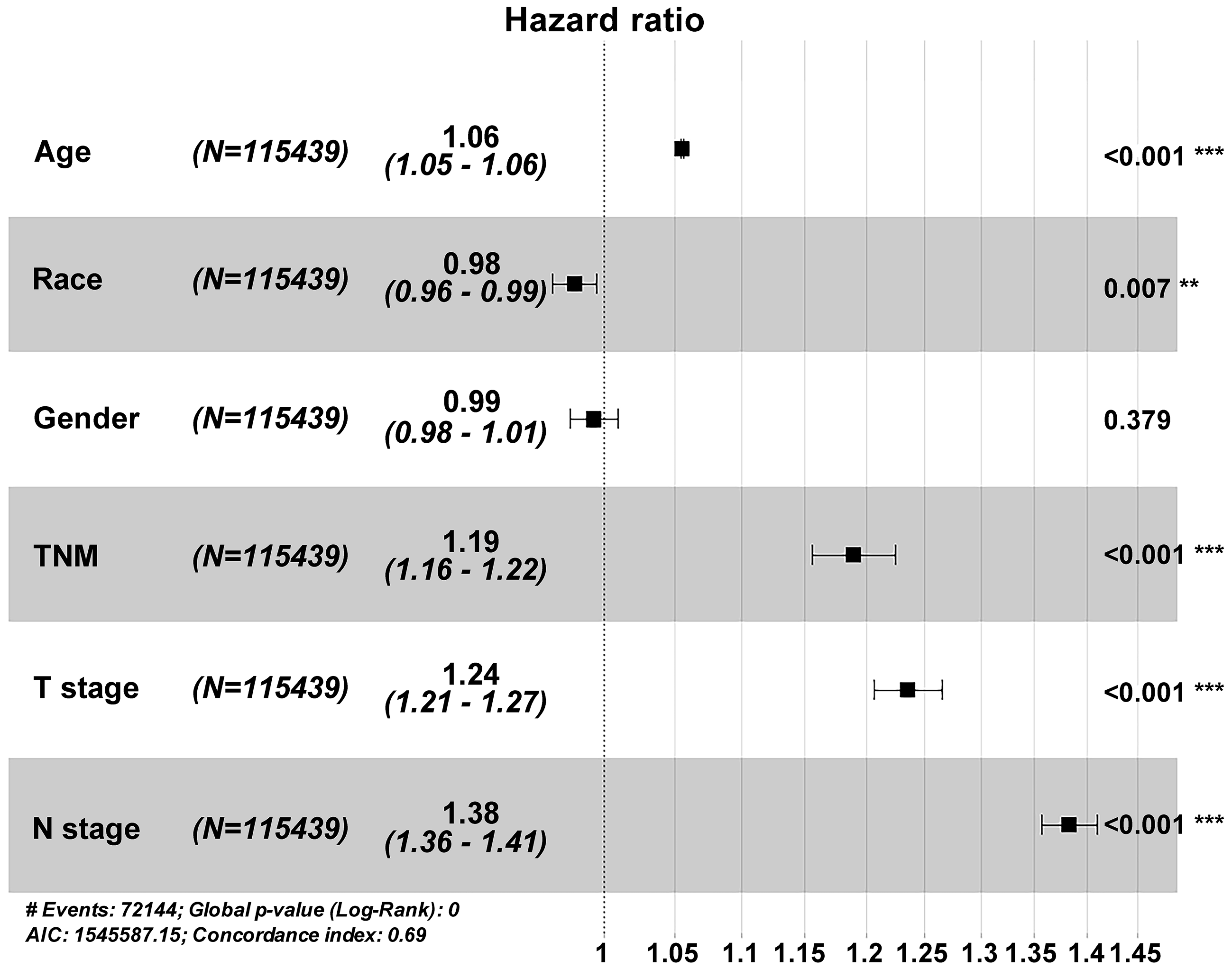

Supplement: Supplementary Figure 2 — Cox analysis for patients with MIBC from the SEER database. [file Image_2.tif]

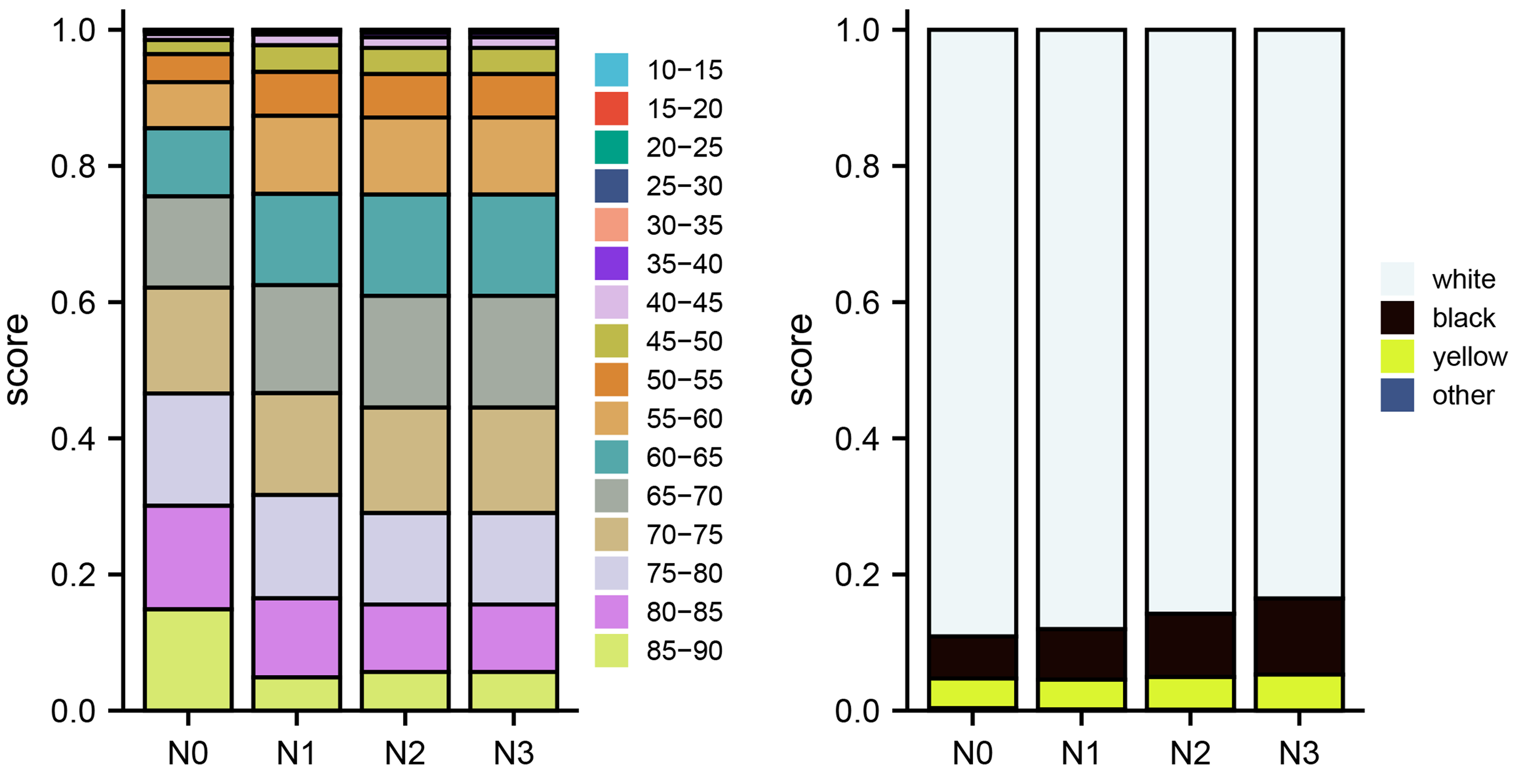

Supplement: Supplementary Figure 3 — The relationship between clinical features and N stage. [file Image_3.tif]

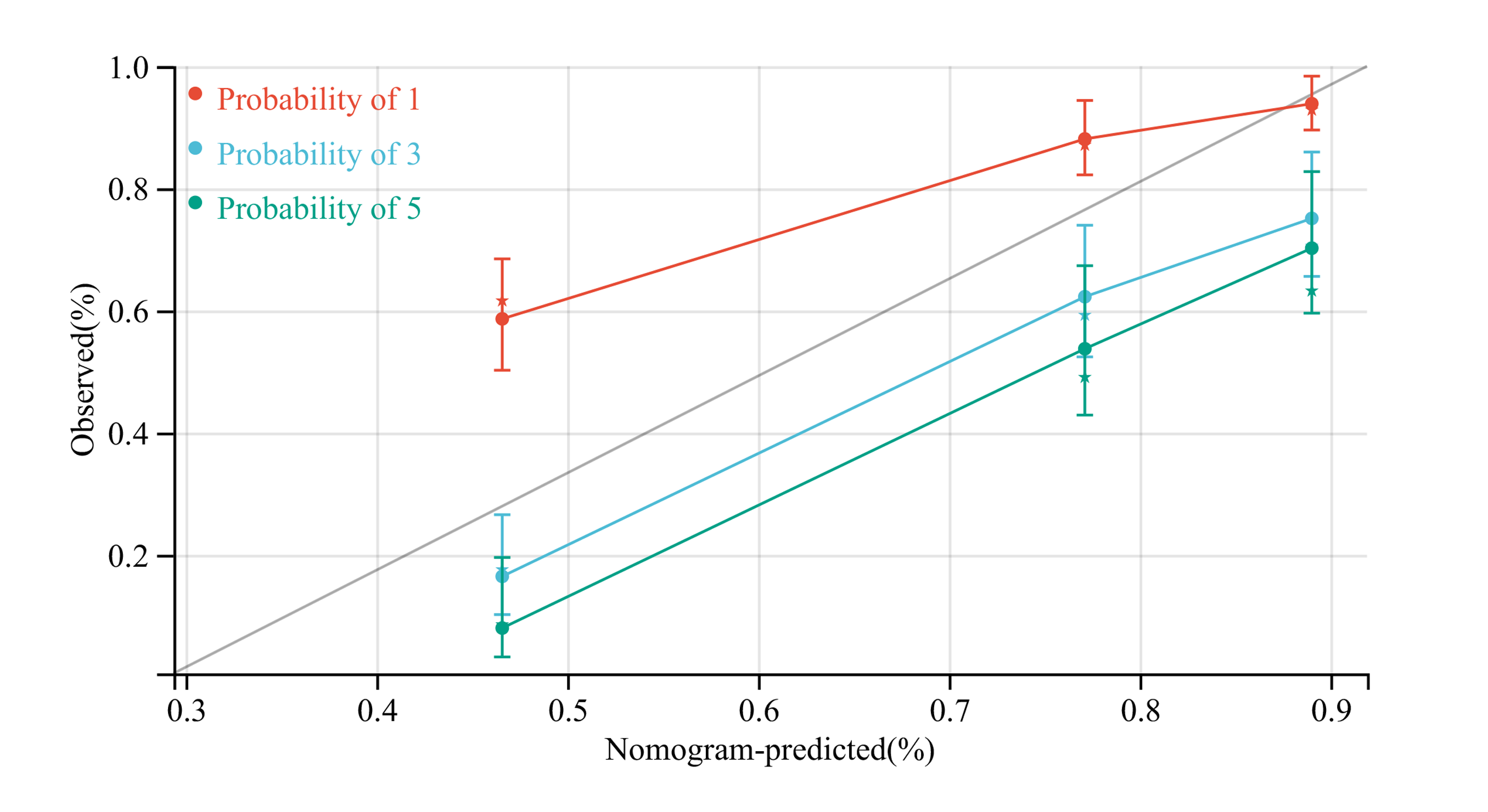

Supplement: Supplementary Figure 4 — The calibration of constructed nomogram. [file Image_4.tif]

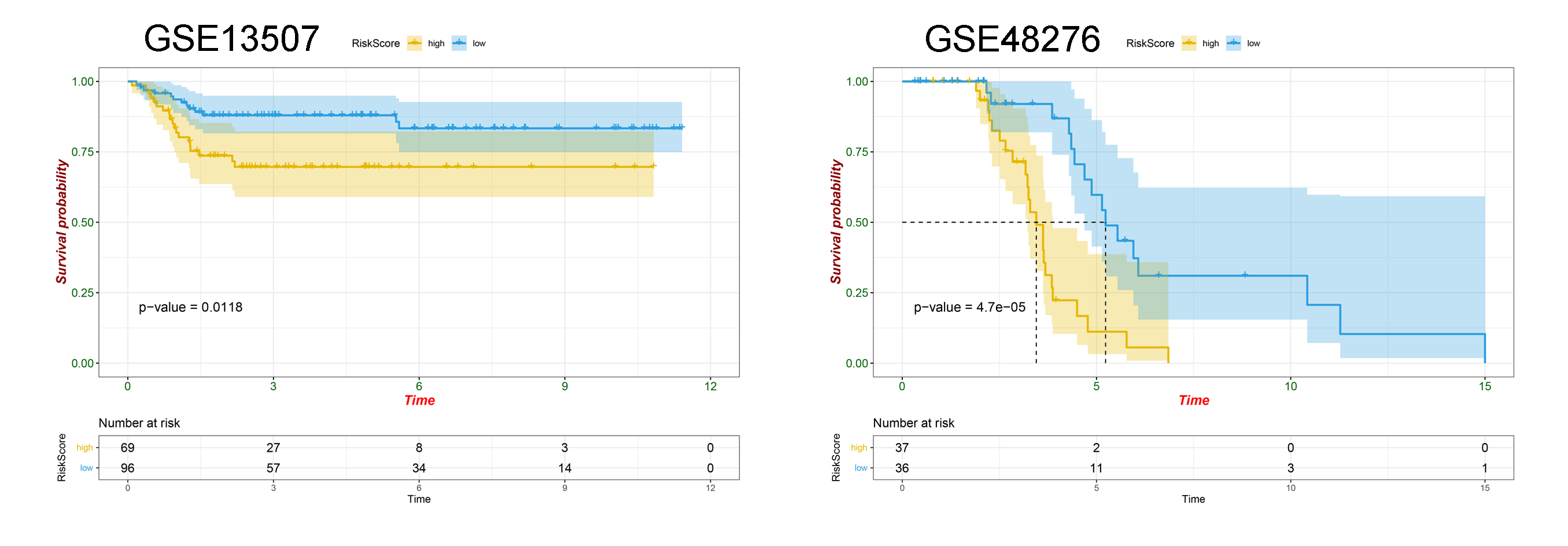

Supplement: Supplementary Figure 5 — The KM plot of risk score in GSE13507 and GSE48276. [file Image_5.tif]

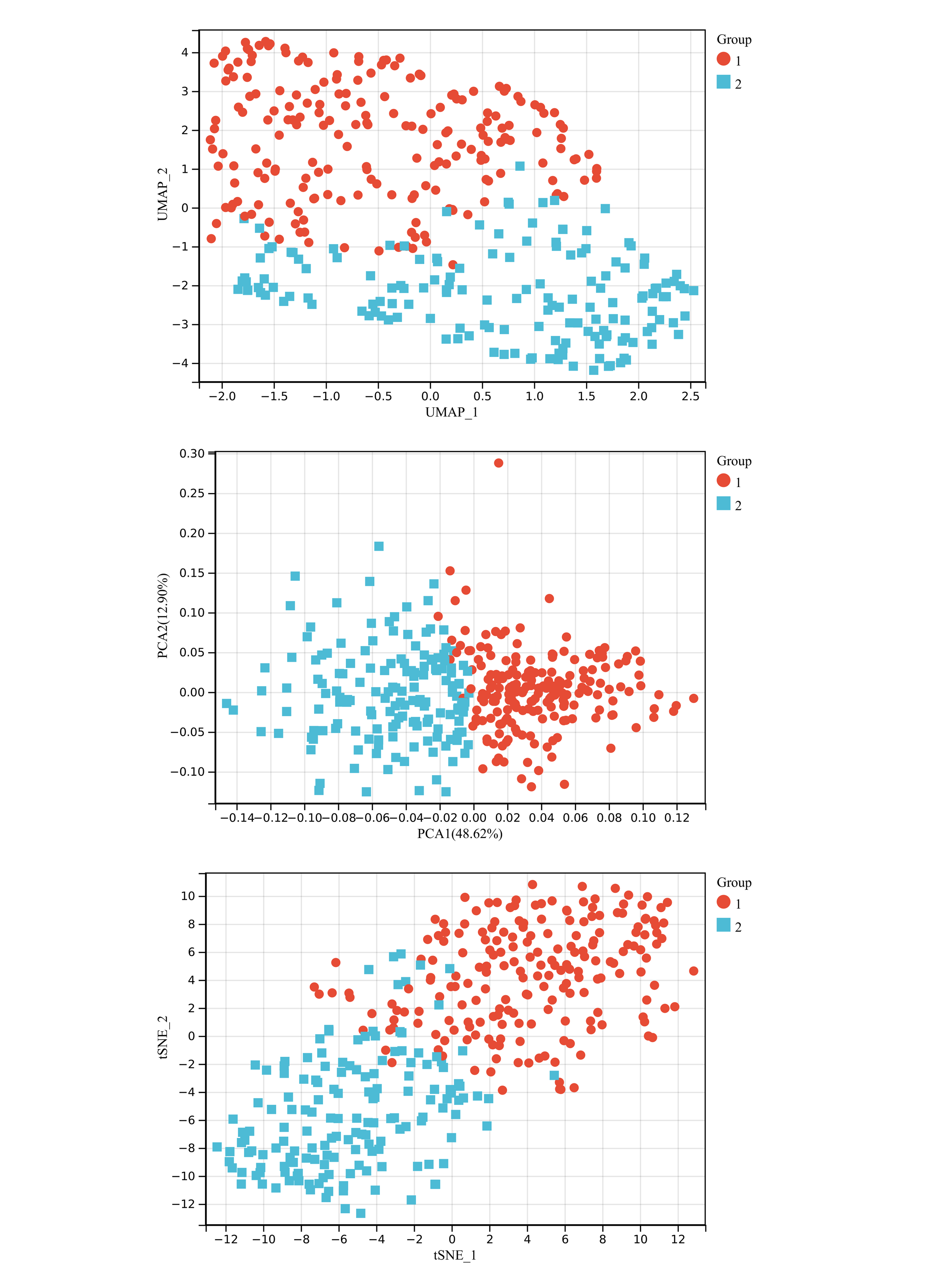

Supplement: Supplementary Figure 6 — The UMAP/PCA/tSNE reduction methods to re-evaluate the cluster. [file Image_6.tif]

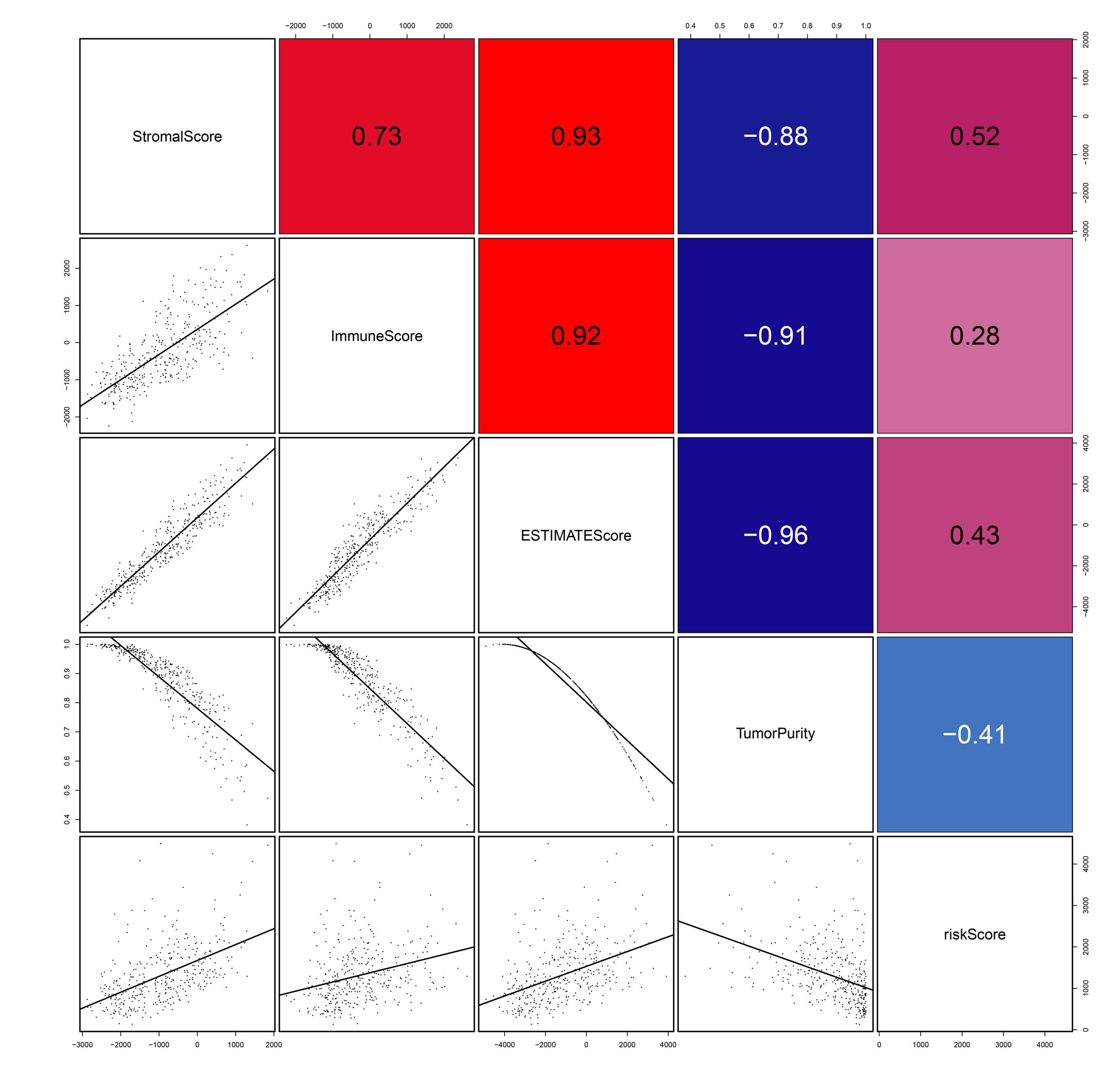

Supplement: Supplementary Figure 7 — The correlation between risk score and immune scores. [file Image_7.tif]
