# Supplementary material for: Identify and validate RUNX2 and LAMA2 as novel prognostic signatures and correlate with immune infiltrates in bladder cancer
Source: Front Oncol. 2023 Jul 13;13:1191398. doi: 10.3389/fonc.2023.1191398 (PMC10373733; doi:10.3389/fonc.2023.1191398)
Supplement: Supplementary Table 1 — The CoefficientmRNA of 19 prognostic biomarkers and the genelist of 45 enrolled genes. [file Table_1.doc]

| **Enrolled genes** |  | **Gene** | **Coef** |
| --- | --- | --- | --- |
| ANK2 | INHBA | C1QTNF6 | 0.107024304 |
| ANPEP | ISLR | DAB2 | -0.015605246 |
| C1QTNF6 | LAMA2 | ESD | 0.41554692 |
| CAPG | LGALS3 | FKBP10 | 0.089377846 |
| COL6A2 | MFAP5 | GAD1 | -0.05537336 |
| COL6A3 | MGLL | INHBA | -0.02558881 |
| CPM | MMP23B | LAMA2 | 0.179585371 |
| DAB2 | MPPED1 | LGALS3 | 0.024405351 |
| DCN | NRP2 | MPPED1 | -0.042099434 |
| DKK3 | NRXN2 | OLFML3 | -0.057826229 |
| ESD | OLFML3 | PCOLCE | -0.112431751 |
| FBN1 | PCOLCE | RASD1 | 0.022977495 |
| FBXL7 | PDGFRA | RGS12 | -0.086682389 |
| FGF1 | PLXNA4 | RUNX2 | 0.105164105 |
| FKBP10 | POSTN | TIMP2 | -0.004351608 |
| GAD1 | RASD1 | TMEM151A | 0.023651595 |
| GAS6 | RGS12 | TSSK1B | -0.003221671 |
| GSN | RUNX2 | VAT1 | 0.088315706 |
| HGF | SGCD | VEGFA | -0.056722108 |
| SLIT2 | TSSK1B |  |  |
| TIMP2 | VAT1 |  |  |
| TMEM151A | VEGFA |  |  |
| IL1R1 |  |  |  |
