# Supplementary material for: Identify and validate RUNX2 and LAMA2 as novel prognostic signatures and correlate with immune infiltrates in bladder cancer
Source: Front Oncol. 2023 Jul 13;13:1191398. doi: 10.3389/fonc.2023.1191398 (PMC10373733; doi:10.3389/fonc.2023.1191398)
Supplement: Supplementary Table 2 — The selection of hub genes according to the cytoHubba from Cytoscape. [file Table_2.doc]

| **Node_name** | **MCC** | **DMNC** | **MNC** | **Degree** | **EPC** | **BottleNeck** | **EcCentricity** | **Closeness** | **Radiality** | **Betweenness** | **Stress** | **ClusteringCoefficient** |
| --- | --- | --- | --- | --- | --- | --- | --- | --- | --- | --- | --- | --- |
| **TSSK1B** | 2 | 0 | 1 | 2 | 4.242 | 1 | 0.25 | 6.91667 | 3.86667 | 1.28571 | 4 | 0 |
| **INHBA** | 6 | 0.2842 | 4 | 4 | 7.015 | 2 | 0.33333 | 9 | 4.46667 | 6.92857 | 24 | 0.5 |
| **RASD1** | 3 | 0.30779 | 2 | 3 | 5.726 | 2 | 0.33333 | 8.33333 | 4.33333 | 11.83333 | 26 | 0.33333 |
| **SLIT2** | 12 | 0.28529 | 6 | 6 | 7.823 | 3 | 0.25 | 10.08333 | 4.6 | 18.5303 | 46 | 0.4 |
| **OLFML3** | 3 | 0.30779 | 2 | 3 | 6.088 | 1 | 0.2 | 7.95 | 4.06667 | 1.84848 | 6 | 0.33333 |
| **VAT1** | 1 | 0 | 1 | 1 | 2.147 | 1 | 0.2 | 5.2 | 3.06667 | 0 | 0 | 0 |
| **ESD** | 2 | 0 | 1 | 2 | 4.016 | 2 | 0.25 | 7.25 | 4 | 28 | 52 | 0 |
| **PCOLCE** | 5 | 0.30779 | 2 | 5 | 7.295 | 3 | 0.25 | 9.41667 | 4.46667 | 13.57792 | 42 | 0.1 |
| **FKBP10** | 4 | 0.30779 | 2 | 4 | 6.694 | 1 | 0.25 | 9.08333 | 4.46667 | 15.90476 | 40 | 0.16667 |
| **DAB2** | 10 | 0.32413 | 5 | 5 | 7.661 | 1 | 0.25 | 9.41667 | 4.46667 | 4.48268 | 16 | 0.5 |
| **LGALS3** | 30 | 0.51861 | 5 | 5 | 7.797 | 1 | 0.33333 | 9.66667 | 4.6 | 4.29221 | 22 | 0.8 |
| **TIMP2** | 31 | 0.51861 | 5 | 6 | 8.154 | 1 | 0.33333 | 10.16667 | 4.66667 | 13.4026 | 48 | 0.53333 |
| **RUNX2** | 29 | 0.38039 | 6 | 7 | 8.207 | 6 | 0.33333 | 10.83333 | 4.8 | 62.47403 | 138 | 0.38095 |
| **LAMA2** | 10 | 0.32413 | 5 | 5 | 7.64 | 1 | 0.25 | 9.08333 | 4.33333 | 7.90476 | 28 | 0.5 |
| **HGF** | 38 | 0.43905 | 7 | 7 | 8.599 | 3 | 0.33333 | 10.66667 | 4.73333 | 14.33117 | 52 | 0.57143 |
| **VEGFA** | 38 | 0.31026 | 9 | 9 | 8.808 | 1 | 0.33333 | 11.83333 | 4.93333 | 43.20346 | 110 | 0.36111 |
