# Supplementary material for: Identify and validate RUNX2 and LAMA2 as novel prognostic signatures and correlate with immune infiltrates in bladder cancer
Source: Front Oncol. 2023 Jul 13;13:1191398. doi: 10.3389/fonc.2023.1191398 (PMC10373733; doi:10.3389/fonc.2023.1191398)
Supplement: Supplementary Table 3 — The sequences of primers and siRNA used in this study. [file Table_3.doc]

| **The sequences of primers for PCR (5′→3′)** | |
| --- | --- |
| LAMA2 | Forward: CTGCCAACGCTGAGAAACTTG |
| LAMA2 | Reverse: ACATTCTCCAGGGAACATCCT |
| RUNX2 | Forward: AGTGCGGTGCAAACTTTCTC |
| RUNX2 | Reverse: TGACTCTGTTGGTCTCGGTG |
| GAPDH | Forward: GTCAGCCGCATCTTCTTT |
| GAPDH | Reverse: CGCCCAATACGACCAAAT |
| **The sequences of siRNAs (5′→3′)** | |
| NC | Sense: UUCUCCGAACGUGUCACGU |
| NC | Antisense: ACGUGACACGUUCGGAGAA |
| si-LAMA2#1 | Sense: GGUAUAAACUGCGAGACAUTT |
| si-LAMA2#1 | Antisense: AUGUCUCGCAGUUUAUACCTT |
| si-LAMA2#2 | Sense: GGUAAUAAUUGUGACCCAATT |
| si-LAMA2#2 | Antisense: UUGGGUCACAAUUAUUACCTT |
| si-RUNX2#1 | Sense: AAGGUUCAACGAUCUGAGAUUUTT |
| si-RUNX2#1 | Antisense: AAAUCUCAGAUCGUUGAACCUUTT |
| si-RUNX2#2 | Sense: GCACUCCAUAUCUCUACUATT |
| si-RUNX2#2 | Antisense: UAGUAGAGAUAUGGAGUGCUG |
